# The Effect of Supplemental Lighting during the Late Gestation Period on Post-Partum Mechanical Properties of Mare and Foal Guard Hair

**DOI:** 10.3390/vetsci11010049

**Published:** 2024-01-22

**Authors:** András Gáspárdy, Gemma Gallagher, Boróka Bartha, Helene Haaland, Sándor György Fekete

**Affiliations:** Institute for Animal Breeding, Nutrition and Laboratory Animal Science, University of Veterinary Medicine Budapest, István Street 2, 1078 Budapest, Hungary; gemma-gallagher@hotmail.com (G.G.); bartha.boro@gmail.com (B.B.); fekete.sandor@univet.hu (S.G.F.)

**Keywords:** advanced breeding season, blue light-treated mare, guard hair quality, tensile strength, maximum force

## Abstract

**Simple Summary:**

The equine species are seasonal breeders whose physiological breeding season extends from March to May. However, there is a significant demand for foals born early in the year (January–February), which has led to an artificially induced breeding season. This study investigates Thoroughbred mares exposed to artificial blue light at the end of gestation. The aim is to investigate the consequences of this treatment on maternal and filial hair properties at the time of parturition. Sixty mares and their 60 foals were selected for this investigation. Guard hair samples were collected from the shoulder region within 12 h of parturition. Results revealed that the foals of the light-treated mares developed significantly shorter hair than the foals of the control group. The tensile strength of the foal hairs of the light-treated mares was significantly lower than that of the control foals. The hair of the control foals was more elastic than that of the treated mares’ foals. In summary, blue light treatment affects the hair, making it thinner and weaker in both mares and their foals.

**Abstract:**

This study investigates Thoroughbred mares exposed or not to supplemental blue light at the end of the gestation. Sixty mares and their 60 foals were selected for the investigation. Guard hair samples were collected from the shoulder just after (within 12 h) the parturition or birth. The foals of the light-treated mares developed significantly (*p* < 0.05) shorter hair than those of the control mares. A general effect of light treatment on basal hair diameter thinning could be demonstrated (*p* < 0.005). The maximum force of hair samples of light-treated mares and foals (0.098 and 0.085 N, respectively) was significantly lower than that of the control (0.272 and 0.178 N, respectively). The tensile strength (82.2 N/mm^2^) of the foal hair samples of the light-treated mares was significantly lower than that of the control foals (121.6 N/mm^2^). Although no significant difference was found in the elongation (ΔL), the hair of the control animals (mares and foals together) was more elastic than that of the treated animals (335 vs. 262 μm). In conclusion, the supplemental blue light treatment of the pregnant mares has a decreasing effect on both mares and their foals on the mechanical properties of the hair, making it shorter, thinner, and weaker.

## 1. Introduction

The equine species are seasonal breeders and will start their breeding season between March and May. Setting 1 January as the universal birth date has created a high demand for foals born early in the year (January–February) to produce mature yearlings and more developed 2-year-old racehorses. Previous research has shown that annual earnings are significantly higher for horses born in January–February than for horses born in April–June. This fuels an artificially advanced breeding season [1].

For breeders to satisfy competition timelines, it is necessary to meet the official starting date of the breeding season on 15 February. Thus, to meet this starting date, a standard management tool on commercial horse breeding farms is to expose the mares to artificial light in order to advance seasonal reproductive activity [2]. These artificial light programs mimic the natural processes of increased hours of daylight that lead to early ovulation and, thus, the beginning of the reproductive active period. Beginning the administration of artificial light in early December will allow the breeders to meet the industry-imposed breeding timelines and economic goals. Considering the relation between light, melatonin, and hair development, studies (e.g., [2]) aimed to investigate what impact, if any, this unnatural photoperiod during the final months of gestation could have on maternal and filial guard hair properties at the time of parturition.

### 1.1. Reproduction Cycle of the Mare

The horse mare is a seasonal polyoestrous animal, manifested by a circannual endogenous rhythm influenced by exogenous factors such as nutrition, body condition, environmental temperature, and photoperiodic changes. The mare is a long-day breeder, which means that her natural physiological breeding season is stimulated by increasing hours of daylight, associated with spring, and the related reduction of melatonin secretion. The natural breeding season of the mare will, therefore, extend from approximately April to September in the northern hemisphere [3].

Photoperiod is the primary cue that triggers the mare’s reproductive system to become active at the appropriate time of the year [4]. Photoperiodism is the ability of an organism to adjust its physiology, oestrous cyclicity, and behaviour to seasonal changes in the natural environment according to the length of daylight (photoperiod) [5]. These photoperiodic changes are translated into an endocrine signal in the pineal gland, where melatonin is secreted during the hours of darkness [6]. During long days, the inhibitory action of melatonin of the mare´s reproductive axis is lifted, and increased gonadotropin-releasing hormone pulse frequency stimulates the anterior pituitary to release follicle-stimulating hormone and luteinizing hormone, which, in turn, will promote the growth, development, and ovulation of ovarian follicles [7].

### 1.2. Hair Development of Foetus and Foal

Hair growth is cyclic, with hair-producing follicles moving between active (anagen) and quiescent (telogen) phases. The main controls of hair follicle cycling among mammalian species are the same. Prolactin is one of the essential regulators [8]. The pelage cycle is influenced by the seasons, but in Equidae, the donkey, opposite to the horse, does not produce a winter coat. On the contrary, the horses’ pelage shows important circannual changes. The winter hairs are thicker than summer [9]. Shedding from the long hair follicles in horses (e.g., mane) has a peculiar cycle: these cycles are not influenced by light or temperature [10].

Foetal hair starts to grow at around 270 days of gestation. Post-partum hair growth is approximately 1 cm per month in foal [11], and a similar intensity of growth can be observed in adult humans [12].

By measuring cortisol and androsterone derivates in the hair samples collected at birth, it is possible to assess prenatal adrenal activity and the effect of stressful events on neonatal hair development. The hair of the foal reflects not only the mother’s endocrine events but perhaps its glucocorticoid concentration, which serves as a potential biomarker of prenatal diseases [13]. Therefore, hair samples collected immediately after the birth reflect hormonal influences in the last third of pregnancy.

### 1.3. Artificial Light Administration

The use of an artificially extended photoperiod is a widespread method in the thoroughbred industry, and at the same time, more people are paying attention to the investigation of the consequences of this unnatural breeding. A common observation of mares exposed to an artificially extended photoperiod is the early shedding of the heavier winter coat [2].

The use of artificial light to alter the natural physiological breeding season in mares has become a common practice within the equine industry as it has proven helpful in both pre-partum and post-partum mares. Collery et al. (2023) [14] proved core circadian clock genes’ rhythmicity in the hair follicles, which is strongly influenced by stable lighting in horses. Research has proven that a photoperiod regiment of 16 h of light and 8 h of darkness provided by a 100 W light bulb in a 12-foot by 12-foot stall is sufficient to achieve advanced seasonal active reproductivity [15]. It is described by Gooley et al. (2013) [16] that the level of light required for melatonin inhibition is not related to vision, but to the level of light required to stimulate the suprachiasmatic nuclei (SCN). A recent study by Nolan et al. (2017) [2] has shown that low-intensity blue light (468 nm) from light-emitting diodes (LED) directed at a single eye can sufficiently suppress melatonin secretion and is an effective way of altering natural breeding season. Supplementary blue light during the shorter winter days stimulates early oestrus and ovulation [17,18].

### 1.4. Effects of an Extended Photoperiod on Coat Condition in Adult Horses and Foals

O’Brian et al. (2020) [19] evaluated the effects of supplemental lighting adjusted to the autumnal equinox and the winter solstice on the coats of adult horses and ponies. They found that extended photoperiod and warmth to stabled horses resulted in shorter and lighter hair coats as well as higher shedding scores over time. Lutzer et al. (2022) [20] investigated the effect of the pre-partum extended day length using a blue LED light mask on one eye on the pregnancy of the mares and the traits of their newborn foals. They stated that mares exposed to the long day photoperiod from mid-December onwards foaled earlier than the control mares. Foals born to treatment mares were of lower size and had a significantly shorter hair coat at birth but did not differ in weight from foals born from control pregnancies.

Kunii et al. (2015) [21] and Harada et al. (2015) [22] divided weaned Thoroughbred foals into two groups in each experiment. One group (treatment group) was subjected to an extended photoperiod from the end of December to the beginning of April and May, respectively, while the control group was subjected to natural light alone. The lighting conditions were set to 14.5 h of light and 9.5 h of dark. Coat conditions of the foals were repeatedly evaluated later. There was no significant difference in the coat conditions of the two groups (treatment and control) in January. However, in April, the foals of the treatment group had changed from winter to summer coats (shedding of winter coats). Both studies [21,22] proved that an extended photoperiod would promote the shedding of the winter coat. The study carried out by Kunii et al. (2015) [21] also demonstrated that the early elevation of circulating prolactin observed in the foals is promoted by the extended photoperiod. On this basis, it could be suggested that prolactin may have been involved in the promotion of shedding of the winter coat observed in this study.

According to the study compiled by Nolan et al. (2017) [2], the foals of the light-treated group had significantly lower hair weights than those of the control group. It also showed significant differences in the hair length between the treatment and the control group, demonstrating that the hair of the foals from the treatment groups was significantly shorter than the hair of the foals of the control group.

It is predicted that the foetus is exposed to the rhythms of the mare´s internal milieu, such as temperature and endocrine profiles, including melatonin concentration. Melatonin is one of the few maternal hormones that can cross the placenta unaltered. The plasma melatonin adjusted for the moment of birth in newborn foals turns out to be lower than the maternal concentration (27.63 pg/mL vs. maternal 34.58 pg/mL) [23]. However, they found that supplementary light treatment administered during the end of pregnancy reduced nocturnal melatonin concentration in mares at the time of foaling. Moreover, the foals of these mares were born with significantly lower melatonin levels than control foals.

### 1.5. Aim

This study aimed to evaluate how supplemental lighting, provided for late pregnant Thoroughbred mares during an artificially induced season, could influence the guard hair properties of mothers and newborn progenies by the time of parturition. It was hypothesized that this effect is significant for the mechanical properties of hair, and, like responses to light supplementation in mares, light supplementation also affects the foetal coat growth, supplemental lighting length dependant change in diameter, tensile strength, maximum force, and elongation.

## 2. Materials and Methods

### 2.1. Experimental Design

The study took place from the beginning of January to the end of April in a commercial Thoroughbred stud at 53° N. During the study, all pregnant mares were in pasture during the day, where they grazed ad libitum until 16:00 h, and at night they were in individual stalls. The mares had access to ad libitum hay while in the stalls. In addition, they were fed a Red Mills Stud Cubes feed mix (14% crude protein, 4.5% ether extracts, 10% crude fibre). They were allowed 1 kg of this in the morning and 2–3 kg in the evening. The animals had free access to drinking water. Before the start of the experiment and the separation of the test groups, the pregnant mares were kept together in the same place under the same conditions as the later control group. The individuals of the groups were housed in the same conditions, so they were equally affected by the seasonal changes in temperature.

The supplementary lighting of the treated mares was started 41 days before the expected foaling date, with the aim of allowing the mares to breed again sooner after foaling. For them, a Luminaire light fixture from Equilume Stable Light was installed in each stall. This luminaire supplemented the natural daylight with a high-intensity blue component (468 nm, 80 lux) for 15 h per day (corresponding to the length of the natural day at the end of the experiment). Foals were coded in the processing according to their mother’s lighting group.

The control mares were maintained under light conditions reflecting the natural photoperiod typical for the time of year and the location. For the hours of daylight, the control mares were kept at pasture. During the hours of darkness, they were placed in individual stalls without lights. Natural daytime lengths at the beginning were 7 h 50 min and 14 h 53 min at the end of the study.

The investigation was conducted following Directive 2010/63/EU of the European Parliament and the Council on the Protection of Animals Used for Scientific Purposes. This study is exempt from review by the University of Veterinary Medicine Budapest’s Animal Welfare Committee as they met the following criterion for exemption: ‘The study does not involve euthanizing a living animal or conducting a procedure on a living animal’, where ‘procedure’ is defined as ‘any use, invasive or non-invasive, of an animal for experimental or other scientific purposes, with known or unknown outcome, or educational purposes, which may cause the animal a level of pain, suffering, distress or lasting harm equivalent to, or higher than, that caused by the introduction of a needle under good veterinary practice’ in S.I. No. 543/2012—European Union (Protection of Animals used for Scientific Purposes) Regulations 2012.

### 2.2. Sample Collection and Measuring

One hundred and twenty individuals (60 mares and their 60 born foals) were selected for the investigation. Guard hair samples (including hair root) were collected from the shoulder within 12 h of parturition and stored in labelled bags until processing.

Six representative hairs from the samples of each individual were randomly selected for processing; thus, the evaluation is based on nearly 720 observations for each characteristic (this number is less due to measurement errors and the exclusion of outliers in some samples).

First, the length of the hair was measured, and then, after some preparation, the other properties were measured and calculated. The measured hair samples were then fixed onto a paper frame using two adhesive strips. Once the hair length was between 15 and 20 mm and the standard internal gap of the paper frame was 10 mm, the basal part of the hair shaft was fitted to the bottom point of the frame. This circumstance made it possible to retrospectively observe the final period of hair growth on the most proximal 10 mm segment to the skin. To facilitate microscopic analysis, the paper frame was fixed onto a specially-made slide.

OLYMPUS BX 51M Microscope (Olympus, Austin, TX, U.S.) was used with OLYMPUS Stream Motion Imaging Software (version 1.9.4) to analyse the diameter of each hair at three points: basal point (by the bottom edge of the frame), “medial point” (in the middle of the frame, 5 mm from the basal point), and “apical point” (by the upper edge of the frame, 10 mm from the basal point) in µm. The actual elongation (ΔL) of each hair as well as the maximum force (F_max_) recorded using a Zwick-Z005 tensile testing machine (Zwick, Ulm, Germany) and a 20 N force measuring cell with the Zwick TestXpert 11.0 program were tested. The hair samples were examined at room temperature and 50% relative humidity.

The following traits were determined in the order below:

- Average hair length: The mean length of the hair shaft measured using a millimetre scale;
- Average apical diameter: The mean diameter, in micrometres, of six hairs per mare and foal measured at the upper edge of the frame;
- Average medial diameter: The mean diameter, in micrometres, of six hairs per mare and foal measured in the middle of the frame;
- Average apical–medial change: The mean percentage difference between the apical and medial diameters of six hairs per mare and foal;
- Average basal diameter: The mean diameter, in micrometres, of six hairs per mare and foal measured at the bottom edge of the frame;
- Average apical–basal change: The mean percentage difference between the apical and basal diameters of six hairs per mare and foal;
- Average actual elongation or strain (ΔL): The elongation assessed in the linear elastic region of stress–strain curve, in micrometres, of six hairs per mare and foal;
- Average maximum force (F_max_): The mean maximum force, in Newton (N) with speed for tear testing of 10 mm per minute, of six hairs per mare and foal recorded at the time of the breaking of a given hair;
- Average tensile strength or stress (TS): The mean ratio of the maximum force (F_max_) to the surface area (A) of the narrowest diameter (d), in N/mm^2^ (or MPa), of six hairs per mare and foal (F_max_/A = F_max_/π*(0.5*d)^2^).

### 2.3. Statistical Analysis

The Dixon test was used to test the source data for outliers. During the Dixon test, the ratio of our data (Q) was compared to the critical value (Q_crit_) from the Dixon table. The ratio is defined as the difference between the suspected value and the nearest value divided by the range of the values (Q = rejection quotient). If Q is bigger than Q_crit_, then suspect data is assumed. This was the case for a few values.

Then, the Shapiro–Wilk test of normality was performed on the source data. It gave a seriously significant (*p* < 0.001) result in one of the hair characteristics. This was the average actual elongation. Thus, the natural logarithm transformation (ΔL → eLOG ΔL) of the raw data of elongation was achieved to normalize it. The basic statistics of the investigated traits are shown in Table 1, together with the results of the Shapiro–Wilk test (*p*-value). For further statistical analysis, the eLOG average actual elongation was used, among other traits. The mean values of the basic statistics preliminarily show that the foals have longer and coarser guard hairs than their mothers, making them stronger and more resistant.

The characteristics of the hair were evaluated using a single trait general linear model (GLM). Applying this model twice, at first the category (with four classes: control mares, foals of control mares, light-treated mares, and foals of light-treated mares), then the group (with two classes: control and light treated) and the generation (with two classes: mares and foals) were included as fixed effects along with foal sex. The age of the mare, the length of gestation, the days from winter solstice to foaling, the weight of the mare, and the weight of the foal were considered covariates (Table 2).

The description of the covariates is as follows:

Age of the mare: determined from the date of birth and the date of actual foaling, in years.

Length of gestation: calculated from the date of successful mating and the date of actual foaling, in days.

Days from winter solstice to foaling: the time elapsed from December 21st of the previous year to the date of actual foaling, in days.

Weight of mare: the estimated weight of the dam before foaling, in kilograms

Weight of foal: measured on the day of birth, in kilograms.

For traits of normal distribution, the gained results are given as Least Square Mean (LSM), Standard Error of Mean (SE), the percentage difference between the treatment group and the control group, and the upper and lower 95% confidence levels (−95% CI and +95% CI). The geometric mean and the upper and lower 95% bounds of confidence (−95% CI and +95% CI) are presented after the back transforming (eLOG ΔL → BACK ΔL) for the trait (average actual elongation) normalized. Tukey’s HSD post hoc test was used to detect significant differences among category classes.

All the data were prepared and processed using TIBCO Statistica ver. 13 [24].

## 3. Results

As seen in Table 3, the category of animals had a significant effect on the average hair length. Mares had consistently shorter hair than foals (*p* < 0.05), and among foals, the hair of foals born from light-treated mares was demonstrably shorter than that of control foals (*p* < 0.05). These trends were confirmed by both group and generation effects.

Only generation was found to have a significant effect on the average apical diameter of the hair (see category and generation in Table 4). That is, the mothers’ hair is not only shorter but also thinner than that of their offspring at birth. The group effect was not confirmed, i.e., there was no difference between the classes of the group effect around the start of the light treatment (in other words, light treatment 41 days before foaling has not yet resulted in a demonstrable difference between mares). This situation is favourable for the research because it confirms the same initial condition of the mares included in this study.

Regarding the average median diameter, it can also be concluded that there is a proven effect of generation and category on this hair characteristic (Table 5). Mares have thinner hair than foals. In terms of the variation of hair diameter over time, the mares’ hair thinned towards the end of the gestation period, while the foals’ hair thickened. There was no difference between control and light-treated animals (before foaling approximately 20 days and 5 mm hair growth, and around 20 days after treatment).

The average apical–medial change confirms the differences between generations and categories (Table 6). It confirms the previous findings that mares have thinning hair and foals have thickening hair towards the end of pregnancy (before foaling approximately 20 days). The thinning was more intense in light-treated mares (−15.4 vs. −11.4%), but this was not confirmed by statistical tests.

The previously observed trend of diameters also continued in the average basal diameter (Table 7). This was manifested in a more considerable significant difference between the diameters of the mother and offspring (41.6 vs. 88.9 μm). Nevertheless, more importantly, there was also a significant difference within the group. Within the category, no verified difference could be detected neither between the mean values of treated and control mares, nor between treated and control foals, despite the growing divergence of these.

The difference between the apical and basal diameters in time around foaling has been confirmed for generations (Table 8). The rate of change was −28.0% in mares and 26.5% in foals. What is essential are the category classes, in the knowledge that the category and group-by-generation interaction were not significant. Accordingly, mare hair thinning was more intense in the treated group (−31.1 vs. −24.6%), and foal hair thickening was less pronounced in the treated group (24.4 vs. 28.6%). These data suggest that light treatment influences hair diameter, it significantly stimulates hair thinning in the mares (see additional GLM *p* = 0.024), and might hinder hair thickening in the foals (see additional GLM *p* = 0.330). The basal hair diameter of mares at foaling is negatively and significantly affected by the time since the winter solstice (see additional GLM *p* = 0.002). This determination may be related to shedding.

In Table 9, neither category, group, nor generation has a proven effect on the average actual elongation, despite the apparently large difference between the control and treated groups being mostly given (335 and 262 μm, respectively). This is explained by the comprehensive measurement ranges with overlap. In any case, the hair of the treated animals seems to be less elastic than that of the control animals.

However, since the standard internal gap of the paper frame was 10 mm (10,000 μm), the obtained values can also be interpreted as percentage values of elongation. So, values of percentage elongation (or specific strain, ε) varied around 3%.

Table 10 shows that the average maximum force is influenced by all three effects. Thus, in terms of group and generation, the hairs of control animals and foals are significantly more resistant than those of light-treated animals and mares, respectively. Regarding the category, there is no difference between control and treated mares. However, there is a significant difference in the average maximum force trait between foals of control and treated mares. It makes us think that the light treatment of the dam affects the quality of the foal’s hair, and weakens it! This phenomenon is confirmed by the significant group-by-generation interaction (*p* = 0.002).

Additionally, this trait is negatively and significantly affected by the mare’s weight (*p* = 0.023, regression coefficient (beta): −0.1681). Perhaps this result should be treated with caution because the weight of the mares was estimated.

At the same time, the average maximum force is positively and significantly affected by the foal’s birth weight (*p* = 0.002, regression coefficient (beta): 0.2135). It suggests that the heavier the newborn foal, the more resistant its coat.

The average tensile strength as a complex indicator is like the previous one. The effect of category and generation was significant for this hair property. Table 11 shows that foals have stronger hair at birth than mares at foaling. It is especially true for foals born to control dams, which is confirmed by the significant group-by-generation interaction (*p* = 0.004). To put it another way, there is evidence of an adverse effect of supplementary light treatment on the hair strength of foals of treated mares.

Here, as before, the foal’s birth weight has a positive and significant effect on this hair property (*p* = 0.013, regression coefficient (beta): 0.2311). In other words, the heavier the foal is, the stronger its hair. The standardized beta coefficient displays the relative contribution of this independent variable to the prediction of the average tensile strength, which is close to 25%.

## 4. Discussion

Compared to the few previous hair studies in the horse, in the present investigation, the hair characteristics were examined in detail, measuring not only the length but also the time-dependent change in diameter, maximum force, tensile strength, and elongation in mares and foals.

As a consequence of the additional illumination, a shortening in hair length was observed, in agreement with previous observations. This effect was statistically significant among the newborns. The findings of this study strengthen the results of the previous work of Nolan et al. (2017) [2], where the hairs of foals born of mares treated with low-intensity, short-wavelength blue light to a single eye proved to be shorter and lighter than the hairs of foals born of control mares. An extended photoperiod is indeed to be responsible for the delivery of a circadian signal to the developing foetus, either by melatonin crossing the placenta to then bind to the foetal melatonin receptors, or indirectly as a response to the changing endocrine values of the mare. In the current study, there was a 10% difference in the brevity of the hair in favour of the foals of the mares treated with supplementary light. It was about 25% in the study of Nolan et al. (2017) [2]. O’Brian et al. (2020) [19] controlled the effectiveness of extended light and warmth applied one month after the winter solstice. To their experience, the treatment did not significantly decrease the hair length or hair weight in indoor or outdoor living Connemara ponies, as has been the case in this processing. In contrast, in the current study, the administration of an extended photoperiod after the winter solstice significantly affected the hair length in the foals.

In addition to the absolute values of the diameter measured at different locations on the hair, the variation of the diameters over time is even more revealing. The degree of change of the apical and basal diameters significantly differed for generations (−28.0% in mares vs. 26.5% in foals). According to the treatment, the mares’ hair thinning was more intense in the treated group (−31.1 vs. −24.6%), and the foals’ hair thickening was less pronounced in the treated group (24.4 vs. 28.6%), and significant for pair-wise statistical comparisons. These data suggest that light treatment specifically influences hair diameter at foaling, causing the mares’ hair thinning, and, at the same time, preventing the foals’ hair thickening. The current study demonstrates that the hair samples of the treated mares’ foals are weaker, and the hair thickening process is less intensive than in the case of the control group as a response to the significant effect of an artificially extended photoperiod. Examination of the hair diameter complements previous knowledge. According to this, the guard hair of the light-supplemented individuals is lighter-weighted than that of the controls not only because it is shorter [2,25], but also because it is thinner.

The effects of other light-producing hormones, such as melatonin, IGF-2, and prolactin on the gestation and hair properties are also presumed.

In the current study, the basal hair diameter of mares at foaling is negatively and significantly affected by the time since the winter solstice. So, there is a fluent thinning of the mare´s hair before shedding, which is consistent with the finding of DeBoer et al. (2023) [26]. However, a more intense reduction is observed in the case of mares treated with supplementary blue light.

In our study, mares were exposed to light supplementation for 41 days at the beginning of the year, when hair growth is faster (almost 10 mm per January and February) than in other periods of the year [27]. Measuring the diameter in three places made it possible to evaluate the effect of additional light during this pre-foaling period. The trends established here are acceptable from a professional point of view. A significant difference between the treatment groups was confirmed in the change in the basal diameter. That is, at least one month of light treatment leads to hair thinning. There were more significant differences in average maximum force between foals than between mares. During the test of average maximum force, the hairs of the foals from the treatment group measured a lower (35%) average maximum force than that of the foals of control mares.

Also, the average actual elongation of the hairs of foals from the treatment group measured lower values (22%) than those of the control mares. The hair elongation of the Thoroughbred animals we found (3%) falls short of the values (52 and 46%) established in other breeds (Konik and Hucul, respectively) [28]. It is conceivable that our lower value is partly due to the normalization of the basic data.

The tensile strength is a complex parameter, too: the hair of the foals born from the control (untreated) mares is strong, and the blue light-treated mares’ foals have weaker hair than their control counter partner. It can be stated that the dam’s light treatment reduced the hair development of their offspring. In our processes, the tensile strength was around 100 N/mm^2^. Other sources report similar or slightly higher values for coarser mane [28] and tail [29,30] hairs.

The foals’ birth weight had a positive and significant effect on average maximum force and average tensile strength, which means that the heavier, better developed the foal is, the stronger its hair. Lutzer et al. (2022) [25] studied the effect of blue light treatment of pregnant mares on the development of their offspring. Although the foals had a lower height at withers, lower hair weight, and length, the critical blood parameters did not differ from that of the control foals. A month after the birth, there was no difference in the pelage of the blue light-treated and control mares’ foals. Owing to the compensatory growth, at one year old, even the size (height at withers) of the foals born from light-treated dams attained that of the controls.

The shortening and thinning of the hair in light-treated animals will undoubtedly affect the density and depth of the coat and affect other values of the coat. It may be worthwhile to investigate the thermal insulation value and heat loss at various ambient temperatures and wind speeds of the hair coat of treated animals.

## 5. Conclusions

The present study suggests that by administering low-intensity, short-wavelength blue light following the winter solstice, during the last 41 days of the pregnancy to the pre-partum mare, the hair properties of the foal were significantly influenced. The length and the diameter of the hair decreased, and its maximum force and tensile strength reduced.

## Figures and Tables

**Table 1:** Basic statistics of traits investigated with results of Shapiro–Wilk test (*p*-value).

Trait	n Mares/ Foals	Mean	CV%	*p*-Value
Average hair length, mm	55	16.62	11.6	0.3233
	59	20.89	15.5	0.0735
Average apical diameter, μm	55	57.86	10.0	0.6227
	59	71.11	14.3	0.5993
Average medial diameter, μm	54	50.23	14.6	0.3142
	58	85.92	13.9	0.0390
Average apical–medial change, %	54	−13.36	−54.5	0.2690
	58	21.24	43.9	0.5824
Average basal diameter, μm	54	41.84	19.7	0.9942
	58	89.32	11.3	0.1393
Average apical–basal change, %	54	−27.85	−40.6	0.0572
	58	26.67	46.2	0.0487
Average actual elongation (ΔL), μm	49	486.6	91.0	<0.001
	56	495.8	80.7	<0.001
eLOG average actual elongation (eLOG ΔL)	49	4.67	10.3	0.0748
	56	5.67	13.7	0.6772
Average maximum force (F_max_), N	55	0.0909	31.8	0.1334
	58	0.2256	44.6	0.3904
Average tensile strength (TS), N/mm^2^	54	67.24	24.4	0.1848
	58	101.38	57.2	0.0768

n: number of individuals, CV%: coefficient of variation.

**Table 2:** Basic statistics of covariates.

Covariate	Mean	CV%
Age of the mare, years	8.5	32.88
Length of gestation, days	350.0	3.58
Days from winter solstice to foaling, days	86.3	23.11
Weight of mare, kg	588.2 kg	7.96
Weight of foal, kg	55.5 kg	12.11

CV%: coefficient of variation.

**Table 3:** Results average hair length (mm).

Effect	n	LSM	SE	−95% CI	+95% CI
Category (*p* < 0.001):					
Control mares	28	17.0 ^a^	0.51	16.0	18.1
Foals of control mares	31	21.8 ^c^	0.50	20.9	22.8
Light-treated mares	27	16.2 ^a^	0.53	15.1	17.2
Foals of treated mares	28	19.8 ^b^	0.52	18.8	20.8
Group (*p* = 0.011):					
Control animals	59	19.4 ^b^	0.37	18.7	20.2
Treatment-related animals	55	18.0 ^a^	0.38	17.2	18.7
Generation (*p* < 0.001):					
Mares	55	16.6 ^a^	0.36	15.9	17.3
Foals	59	20.8 ^b^	0.34	20.1	21.5

^a–c^—different subscribed letters show significant differences (Tukey’s HSD post hoc test *p* < 0.05). There is no group-by-generation interaction (*p* = 0.245). Other effects were not significant (*p* > 0.05).

**Table 4:** Results of average apical diameter (10 mm from the basal point, μm).

Effect	n	LSM	SE	−95% CI	+95% CI
Category (*p* < 0.001):					
Control mares	28	58.5 ^a^	1.66	55.2	61.8
Foals of control mares	30	71.7 ^b^	1.66	68.4	75.0
Light-treated mares	26	56.9 ^a^	1.74	53.4	60.3
Foals of treated mares	28	70.1 ^b^	1.70	66.7	73.4
Group (*p* = 0.381):					
Control animals	58	65.0	1.21	62.7	67.5
Treatment-related animals	54	63.5	1.26	61.0	66.0
Generation (*p* < 0.001):					
Mares	54	57.7 ^a^	1.17	55.4	60.0
Foals	58	70.9 ^b^	1.14	68.6	73.1

^a,b^—different subscribed letters show significant differences (Tukey’s HSD post hoc test *p* < 0.05). There is no group-by-generation interaction (*p* = 0.993). Other effects were not significant (*p* > 0.05).

**Table 5:** Results of average medial diameter (5 mm from the basal point, μm).

Effect	n	LSM	SE	−95% CI	+95% CI
Category (*p* < 0.001):					
Control mares	28	52.1 ^a^	1.93	48.3	55.9
Foals of control mares	30	86.7 ^b^	1.93	82.8	90.5
Light-treated mares	26	48.0 ^a^	2.03	43.9	52.0
Foals of treated mares	28	84.5 ^b^	1.98	80.6	88.5
Group (*p* = 0.153):					
Control animals	58	69.4	1.42	66.6	72.2
Treatment-related animals	54	66.2	1.47	63.3	69.2
Generation (*p* < 0.001):					
Mares	54	50.0 ^a^	1.36	47.3	52.7
Foals	58	85.6 ^b^	1.32	83.0	88.2

^a,b^—different subscribed letters show significant differences (Tukey’s HSD post hoc test *p* < 0.05). There is no group-by-generation interaction (*p* = 0.600). Other effects were not significant (*p* > 0.05).

**Table 6:** Results of average apical–medial change (%).

Effect	n	LSM	SE	−95% CI	+95% CI
Category (*p* < 0.001):					
Control mares	28	−11.4 ^a^	1.60	−14.6	−8.2
Foals of control mares	30	21.2 ^b^	1.61	18.0	24.3
Light-treated mares	26	−15.4 ^a^	1.68	−18.7	−12.1
Foals of treated mares	28	21.2 ^b^	1.65	17.9	24.4
Group (*p* = 0.271):					
Control animals	58	4.9	1.18	2.5	7.2
Treatment-related animals	54	2.9	1.22	0.5	5.3
Generation (*p* < 0.001):					
Mares	54	−13.4 ^a^	1.13	−15.6	−11.2
Foals	58	21.2 ^b^	1.10	19.0	23.3

^a,b^—different subscribed letters show significant differences (Tukey’s HSD post hoc test *p* < 0.05). There is no group-by-generation interaction (*p* = 0.206). Other effects were not significant (*p* > 0.05).

**Table 7:** Results of average basal diameter (at the basal point, μm).

Effect	n	LSM	SE	−95% CI	+95% CI
Category (*p* < 0.001):					
Control mares	28	44.4 ^a^	1.73	41.0	47.8
Foals of control mares	30	91.6 ^b^	1.73	88.2	95.0
Light-treated mares	26	38.8 ^a^	1.82	35.2	42.4
Foals of treated mares	28	86.3 ^b^	1.78	82.7	89.8
Group (*p* = 0.006):					
Control animals	58	68.0 ^b^	1.26	65.5	70.5
Treatment-related animals	54	62.5 ^a^	1.31	59.9	65.2
Generation (*p* < 0.001):					
Mares	54	41.6 ^a^	1.22	39.2	44.0
Foals	58	88.9 ^b^	1.18	86.6	91.3

^a,b^—different subscribed letters show significant differences (Tukey’s HSD post hoc test *p* < 0.05). There is no group-by-generation interaction (*p* = 0.950). Other effects were not significant (*p* > 0.05).

**Table 8:** Results of average apical–basal change (%).

Effect	n	LSM	SE	−95% CI	+95% CI
Category (*p* < 0.001):					
Control mares ^+^	28	−24.6 ^a^	2.28	−29.1	−20.1
Foals of control mares ^++^	30	28.6 ^b^	2.28	24.0	33.1
Light-treated mares ^+^	26	−31.1 ^a^	2.39	−36.1	−26.6
Foals of treated mares ^++^	28	24.4 ^b^	2.34	19.8	29.0
Group (*p* = 0.036):					
Control animals	58	2.0 ^b^	1.67	−1.3	5.3
Treatment-related animals	54	−3.5 ^a^	1.74	−6.9	−0.0
Generation (*p* < 0.001):					
Mares	54	−28.0 ^a^	1.61	−31.1	−24.8
Foals	58	26.5 ^b^	1.56	23.4	29.6

^a,b^—different subscribed letters show significant differences (Tukey’s HSD post hoc test *p* < 0.05). There is no group-by-generation interaction (*p* = 0.563). Other effects were not significant (*p* > 0.05). ^+^—additional GLM restricted to mares (control and treated) computed *p* = 0.024. Where the *p*-value for covariate days from the winter solstice to foaling is 0.002, regression coefficient (beta): −0.4807). ^++^—additional GLM restricted to foals (control and treated) computed *p* = 0.330.

**Table 9:** Results of back-transformed average actual elongation (BACK ΔL, μm).

Effect	n	Geometric Mean	−95% CI	+95% CI
Category (*p* = 0.511):				
Control mares	26	336	252	447
Foals of control mares	28	335	254	442
Light-treated mares	23	264	195	358
Foals of treated mares	28	261	197	346
Group (*p* = 0.132):				
Control animals	54	335	273	412
Treatment-related animals	51	262	212	326
Generation (*p* < 0.964):				
Mares	49	298	243	364
Foals	56	296	245	357

There is no significant group-by-generation interaction (*p* = 0.979). Other effects were also not significant (*p* > 0.05).

**Table 10:** Results of average maximum force (F_max_) (N).

Effect	n	LSM	SE	−95% CI	+95% CI
Category (*p* < 0.001):					
Control mares	29	0.098 ^a^	0.0130	0.072	0.124
Foals of control mares	30	0.272 ^c^	0.0130	0.246	0.297
Light treated mares	26	0.085 ^a^	0.0138	0.057	0.112
Foals of treated mares	28	0.178 ^b^	0.0134	0.152	0.205
Group (*p* < 0.001):					
Control animals	59	0.185 ^b^	0.0095	0.166	0.204
Treatment-related animals	54	0.131 ^a^	0.0100	0.112	0.151
Generation (*p* < 0.001):					
Mares	55	0.091 ^a^	0.0092	0.073	0.110
Foals	58	0.225 ^b^	0.0090	0.207	0.243

^a–c^—different subscribed letters show significant differences (Tukey’s HSD post hoc test *p* < 0.05). The *p*-value for the effect mare’s weight is 0.023, and the regression coefficient (beta) is −0.1681. The *p*-value for the effect foal’s birth weight is 0.002, and the regression coefficient (beta) is 0.2135. There is a significant group-by-generation interaction (*p* = 0.002). Other effects were not significant (*p* > 0.05).

**Table 11:** Results of average tensile strength (TS, N/mm^2^).

Effect	n	LSM	SE	−95% CI	+95% CI
Category (*p* < 0.001):					
Control mares	28	64.9 ^a^	7.899	49.2	80.6
Foals of control mares	30	121.6 ^b^	7.911	106.0	137.3
Light-treated mares	26	70.8 ^a^	8.289	54.4	87.3
Foals of treated mares	28	82.2 ^a^	8.111	66.1	98.3
Group (*p* = 0.062):					
Control animals	58	93.3	5.791	81.8	104.8
Treatment-related animals	54	76.5	6.029	64.6	88.5
Generation (*p* < 0.001):					
Mares	54	67.9 ^a^	5.578	56.8	78.9
Foals	58	101.9 ^b^	5.406	91.2	112.7

^a,b^—different subscribed letters show significant differences (Tukey’s HSD post hoc test *p* < 0.05). The *p*-value for the effect mare’s weight is 0.043, and the regression coefficient (beta) is −0.1956. The *p*-value for the effect foal’s birth weight is 0.013, and the regression coefficient (beta) is 0.2311. There is a significant group-by-generation interaction (*p* = 0.004). Other effects were not significant (*p* > 0.05).

## Data Availability

The data that support the findings of this study are available from the corresponding author upon reasonable request.
